# Communicate and Fuse: How Filamentous Fungi Establish and Maintain an Interconnected Mycelial Network

**DOI:** 10.3389/fmicb.2019.00619

**Published:** 2019-03-29

**Authors:** Monika S. Fischer, N. Louise Glass

**Affiliations:** ^1^Department of Plant and Microbial Biology, University of California, Berkeley, Berkeley CA, United States; ^2^Environmental Genomics and Systems Biology Division, The Lawrence Berkeley National Laboratory, Berkeley, CA, United States

**Keywords:** *Neurospora*, cell fusion, MAP kinase signaling, chemotropic interactions, ROS signaling, STRIPAK

## Abstract

Cell-to-cell communication and cell fusion are fundamental biological processes across the tree of life. Survival is often dependent upon being able to identify nearby individuals and respond appropriately. Communication between genetically different individuals allows for the identification of potential mating partners, symbionts, prey, or predators. In contrast, communication between genetically similar (or identical) individuals is important for mediating the development of multicellular organisms or for coordinating density-dependent behaviors (i.e., quorum sensing). This review describes the molecular and genetic mechanisms that mediate cell-to-cell communication and cell fusion between cells of Ascomycete filamentous fungi, with a focus on *Neurospora crassa*. Filamentous fungi exist as a multicellular, multinuclear network of hyphae, and communication-mediated cell fusion is an important aspect of colony development at each stage of the life cycle. Asexual spore germination occurs in a density-dependent manner. Germinated spores (germlings) avoid cells that are genetically different at specific loci, while chemotropically engaging with cells that share identity at these recognition loci. Germlings with genetic identity at recognition loci undergo cell fusion when in close proximity, a fitness attribute that contributes to more rapid colony establishment. Communication and cell fusion also occur between hyphae in a colony, which are important for reinforcing colony architecture and supporting the development of complex structures such as aerial hyphae and sexual reproductive structures. Over 70 genes have been identified in filamentous fungi (primarily *N. crassa*) that are involved in kind recognition, chemotropic interactions, and cell fusion. While the hypothetical signal(s) and receptor(s) remain to be described, a dynamic molecular signaling network that regulates cell-cell interactions has been revealed, including two conserved MAP-Kinase cascades, a conserved STRIPAK complex, transcription factors, a NOX complex involved in the generation of reactive oxygen species, cell-integrity sensors, actin, components of the secretory pathway, and several other proteins. Together these pathways facilitate the integration of extracellular signals, direct polarized growth, and initiate a transcriptional program that reinforces signaling and prepares cells for downstream processes, such as membrane merger, cell fusion and adaptation to heterokaryon formation.

## Cell-To-Cell Communication is a Fundamental Biological Process

Cells rarely exist alone, which has driven the evolution of diverse mechanisms for identifying and responding to the presence of other nearby cells. Animals have evolved complex organs (i.e., eyes and ears) for sensing and interpreting the surrounding environment. However, the functioning of these organs is dependent on the cells that make up each organ and their ability to accurately sense and communicate this information to other cells, resulting in a coordinated response. For example, when light enters the mammalian eye, rod and cone photoreceptors in retinal cells become activated, which triggers a change in the amount of glutamate that retinal cells release (Soucy et al., 1998). Nearby bipolar cells are sensitive to fluctuations in glutamate, which causes a flux in sodium and calcium cations, which activates nearby ganglion cells that initiate a signal transduction pathway that is ultimately received, integrated, and interpreted by multiple cells in the brain (Soucy et al., 1998; Cruz-Martín et al., 2014).

Cell-to-cell signaling is a fundamental biological process that has been well studied in many organisms besides mammals. For example, there is abundant research detailing the mechanisms of molecular warfare between plants and pathogenic fungi (Dodds and Rathjen, 2010), hormone signaling between plant cells (Jaillais and Chory, 2010), pheromone sensing between gametes during mating in yeast (Merlini et al., 2013), and quorum sensing that mediates coordinated behaviors (i.e., bioluminescence or biofilm formation) among bacteria (Waters and Bassler, 2005). The majority of cell-to-cell signaling research has focused on unraveling the mechanisms that mediate two general methods for cell-to-cell communication; either a density-dependent survey of how many other similar cells are nearby (i.e., quorum sensing), or signaling and sensing between cells that are intrinsically different from each other (i.e., host–pathogen interactions, or during mating). This review describes another type of cell-to-cell communication in which communication occurs between genetically identical cells. While this form of communication is likely important for organisms across the tree of life, it is fundamentally important for the development and functioning of filamentous fungi.

Filamentous fungi exist as a multicellular, multinucleate interconnected network of tube-shaped cells called hyphae. In a hyphal colony, cells engage in cell-to-cell communication and undergo chemotropic growth toward each other until they make physical contact. Once contact is established, both cells will initiate the process of cell fusion, in which cell walls are remodeled, plasma membranes fuse, and ultimately the two cells become one with a shared cytoplasm. This process results in the hallmark interconnected colony associated with filamentous fungi (Buller, 1933; Hickey et al., 2002). Cell fusion between hyphae within an individual colony reinforces network architecture and influences the flow of resources throughout the colony (Hickey et al., 2002; Simonin et al., 2012; Roper et al., 2013, 2015). Hyphal fusion can also occur between fungal colonies, and can result in one of two outcomes. First, two genetically similar colonies can fuse together, resulting in shared resources and collaboration instead of competition (Bastiaans et al., 2015). Second, if two colonies that are genetically dissimilar at non-self recognition loci undergo fusion, the fused cells are compartmentalized and programmed cell death is initiated, which prevents shared cytoplasm from mixing throughout both colonies (Saupe, 2000; Glass and Kaneko, 2003; Gonçalves et al., 2017). In *Neurospora crassa* and most other fungi, there is no nuclear fusion or genetic recombination that occurs as a result of somatic cell fusion. However, some fungi (i.e., *Aspergillus* spp., *Candida* spp., and *Ustilago* spp.) are capable of a low frequency of somatic nuclear fusion following hyphal fusion, during which genetic recombination can occur via mitotic crossing-over events and chromosome loss, the so-called parasexual cycle (Garber and Ruddat, 1992; Bennett and Johnson, 2003; Schoustra et al., 2007). Unlike sexual reproduction, parasexual reproduction does not involve any specialized structures (i.e., gametes), meiosis does not occur, and no fruiting body or specialized spores are formed.

Communication-mediated cell fusion is important throughout the life cycle of most fungi, including Ascomycete fungi, such as *N. crassa*. Somatic cell fusion has been reported in fungi for over 100 years (Meyer, 1902). It is unclear whether species in the Mucoromycota undergo hyphal fusion, but hyphal fusion has been observed between colonies of the Glomeromycete species, *Rhizophagus intraradices* (Croll et al., 2009). Basidiomycete fungi depend on a unique form of hyphal fusion that forms small hyphal bridges around septa called ‘clamp connections,’ which are important for facilitating nuclear movement during mitotic growth and maintaining a tightly regulated dikaryotic state within each cell (Buller, 1922; Read et al., 2010). Hyphal fusion is also important for building the tissue that ultimately defines the fruiting body of both Basidiomycete and Ascomycete fungi (Van der Valk and Marchant, 1978; Read et al., 2010).

## Communication and Cell Fusion in *Neurospora crass*a

**FIGURE 1:**
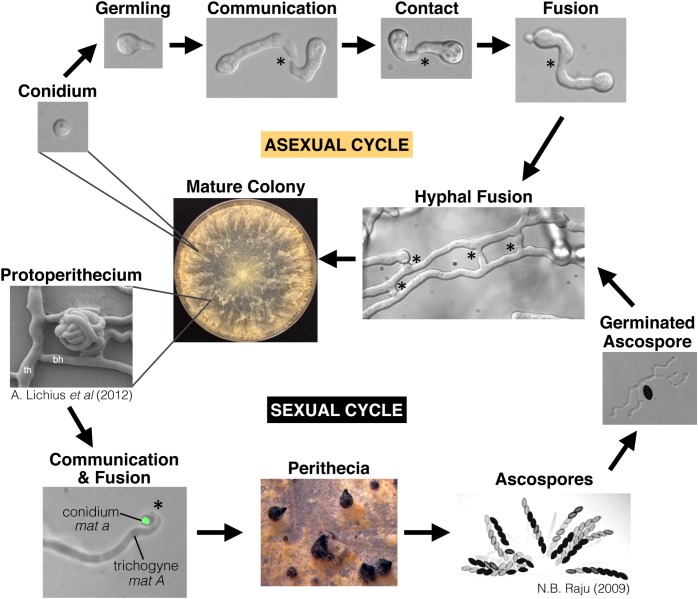
The life-cycle of *Neurospora crassa. N. crassa* is a heterothallic Ascomycete species with a distinct sexual cycle and asexual cycle. Conidia are clonal asexual propagules that can either generate a new colony on their own, or serve as a “male” partner to the “female” trichogyne during mating. The trichogyne is a specialized hypha that emerges from the protoperithecium and chemotropically grows toward a conidium of opposite mating type (shown here expressing H1-GFP). Ascospores are the result of meiosis, which occurs inside the perithecium. This lifecycle specifically highlights chemotropic interactions and cell fusion events. Stars indicate chemotropic interactions and fusion. Germlings, hyphae, and the trichogynes all undergo chemotropism and cell fusion. Protoperithecium image is from Lichius et al. (2012b), and the image showing many ascospores is from Raju (2009), with permission.

*Neurospora crassa* is a well-developed model organism for studying eukaryotic genetics and cell biology, including cell-to-cell communication and cell fusion (Galagan et al., 2003; Colot et al., 2006; Leeder et al., 2011; Herzog et al., 2015). Communication and cell fusion are important at several points throughout the *N. crassa* lifecycle (Figure 1). *N. crassa* is a heterothallic, hermaphroditic species; each colony is capable of producing both male and female structures, but mating only occurs between colonies that encode opposite mating types, *mat A* or *mat a*. Sexual reproduction is initiated by a fertile hypha in the protoperithecium called the trichogyne (“female”) that chemotropically grows toward a “male” cell (conidium or hypha) that is producing the opposite mating pheromone (Kim and Borkovich, 2004, 2006). Upon cell fusion between the trichogyne and the male cell, the male nucleus is transported down the trichogyne to the protoperithecium, where it replicates with female nuclei to form ascogenous hyphae (Backus, 1939). Within ascogenous hyphae, nuclei of opposite mating types undergo one mitotic division and septation to form the crozier. Opposite mating type nuclei in the penultimate cell of the crozier undergo karyogamy, meiosis, and ultimately becomes an ascus containing the eight meiotic ascospores (Raju, 1980; Fleißner et al., 2008b). This process is replicated many times, resulting in hundreds of asci within a single fruiting body.

Communication and fusion also occur during the vegetative phase of the *N. crassa* lifecycle. *N. crassa*, like many Ascomycete species, produces large quantities of clonal, asexual spores (conidia) that are easily dispersed by wind. When these genetically identical conidia land on a suitable substrate they will germinate and chemotropically grow toward each other to undergo cell fusion. Germinated conidia are called germlings. If non-clonal germlings are genetically different at loci termed “determinant of communication” or *doc*, chemotropic interactions are aborted and cell fusion frequency is drastically reduced (Heller et al., 2016). To prove that germling interactions are truly chemotropic, germlings were moved via optical tweezers; germlings re-oriented to maintain growth toward their communication partners (Wright et al., 2007). Lastly, new protein synthesis is not required for chemotropic interactions in *N. crassa* (Fleissner et al., 2009).

As a fungal colony continues to grow, hyphae within it undergo fusion to make the interconnected mycelial network via chemotropic interactions and cell fusion, which is mechanistically equivalent to the process of germling fusion. In fact, mutants that are defective at germling fusion are almost always also defective in hyphal fusion (Fleißner et al., 2008b; Fu et al., 2011). Fusion mutants are also almost always defective at producing extended aerial hyphae and developing female reproductive structures (protoperithecia), indicating that hyphal fusion may be important for supporting the development of these complex structures. However, there is no correlation between hyphal fusion and conidia production, which occurs on aerial hyphae (Li, 2005; Lichius et al., 2012a). Germling and hyphal fusion in *N. crassa* occurs between genetically similar cells and results in cytoplasmic mixing, but nuclei remain intact and there is no genetic recombination (Roper et al., 2011).

Since the discovery of the first hyphal anastomosis mutant, *ham-1* (also called *soft*) (Wilson and Dempsey, 1999), more than 70 genes have been identified that are involved in the process of communication and/or fusion. Broadly speaking, the process of germling/hyphal communication and fusion is multi-faceted. Germlings and fusion-hyphae produce, secrete, and sense a signaling molecule. To achieve chemotropic growth, the signal-sensing machinery is likely integrated with the machinery that directs polarized growth. Once interacting germlings make physical contact, they begin the process of cell fusion, which includes the breakdown of the cell wall, remodeling plasma membranes, and ultimately mixing of cytoplasm (Hickey et al., 2002; Goryachev et al., 2012). A pre-fusion checkpoint reinforces communication with genetically similar individuals and blocks communication with genetically dissimilar individuals (Heller et al., 2016). If two genetically dissimilar individuals do fuse, there is a post-fusion non-self recognition checkpoint mediated by heterokaryon incompatibility (*het*) loci (Daskalov et al., 2017). A recently fused cell will immediately induce cell death if fusion occurs between two cells that differ in specificity at *het* loci. While death is inherently costly, it may be an adaptive behavior to stop the spread of mycoviruses or cheater genotypes within a colony (Glass and Kaneko, 2003). Alternatively, in the plant pathogenic fungus *Colletotrichum lindemuthianum*, it has been hypothesized that temporary suppression of heterokaryon incompatibility may facilitate genetic diversification via horizontal gene transfer (Ishikawa et al., 2012).

## The Cell Wall Integrity Pathway

The Cell Wall Integrity (CWI) pathway is composed of cell wall sensors that transduce information through a Mitogen Activated Protein Kinase (MAPK) cascade, which is a central signaling complex conserved across fungi. The ascomycete yeast, *Saccharomyces cerevisiae*, has five different cell wall sensors that activate the CWI pathway (Rodicio and Heinisch, 2010). A screen of mutants encoding cell wall proteins in *N. crassa* identified two genes (*wsc-1* and *ham-7*) that encode GPI-anchored cell wall proteins that are required for maintenance of the cell wall, resistance to cell wall stress, and phosphorylation of MAK-1 (Maddi et al., 2012). Both the *Δwsc-1* and *Δham-7* mutants have pleiotropic phenotypes, but only the *Δham-7* mutant is defective at germling and hyphal fusion (Maddi et al., 2012). There are likely more cell wall sensors that activate the MAK-1 pathway that have not yet been identified due to functional redundancy or mutant lethality.

The core conserved component of the CWI pathway is a MAPK cascade, which consists of MIK-1 (MAPKKK), MEK-1 (MAPKK), and MAK-1 (MAPK). Scaffold proteins often play an important role in regulating or buffering MAPK pathways, although a scaffold protein is not always necessary for pathway function. In *S. cerevisiae*, Spa2p acts as a scaffold that links the terminal CWI pathway MAPKK and MAPK to the rest of the polarisome, which coordinates polarized growth (van Drogen and Peter, 2002). In *N. crassa*, the *spa-2* gene is dispensable for cell fusion, but necessary for polarized growth, and fluorescently labeled SPA-2 localizes to hyphal tips, germ-tube tips, septa, and sites of cell fusion (Araujo-Palomares et al., 2009; Lichius et al., 2012b). While the *N. crassa* SPA-2 protein is analogous to Spa2p in *S. cerevisiae*, it remains unclear if the *N. crassa* SPA-2 plays a direct roll in MAPK signaling or cell-to-cell communication. In contrast, the *soft* gene (also named *ham-1*) encodes a potential scaffold protein that physically interacts with MIK-1 and MEK-1 in both *N. crassa* and the related ascomycete species, *Sordaria macrospora* (Teichert et al., 2014; Weichert et al., 2016). During chemotropic interactions between genetically identical cells, the SOFT protein shows a dynamic localization pattern in which fluorescently tagged SOFT will assemble into distinct puncta focused at the cell periphery of the germ tube tip in communicating germlings [also called Conidial Anastomosis Tubes (CATs)] (Roca et al., 2005; Fleissner et al., 2009). These SOFT complexes remain assembled at the germ tube tip for roughly 4 min, after which they disassemble and become diffuse in the cytoplasm. Approximately 4 min later, SOFT complexes re-assemble at the germ tube tip. These roughly 4 min SOFT-complex oscillations of assembly and disassembly continue for the duration of chemotropic growth between two communicating germlings or hyphae undergoing chemotropic interactions (Fleissner et al., 2009). In contrast, the MAK-1 protein does not have a dynamic pattern of localization during chemotropic interactions, but localizes to germ tube tips at the site of fusion (Dettmann et al., 2013; Weichert et al., 2016). SOFT physically interacts with MIK-1 and MEK-1, but not MAK-1 (Teichert et al., 2014; Weichert et al., 2016). In *S. macrospora*, the *soft*-ortholog (PRO40) also directly interacts with an upstream activator, PKC1, and indirectly interacts with a Rho GTPase, RHO1. There are orthologs of *rho1* and *pkc1* in *N. crassa*, but there is no available *Δrho-1* mutant, and the *pkc-1* deletion is lethal (Park et al., 2011). Thus it remains unclear if *pkc-1* and *rho-1* are important for the CWI pathway in *N. crassa* or whether these proteins play a role in cell fusion.

Canonical MAPK cascades generally lead to a change in transcription. In *S. cerevisiae*, the CWI pathway regulates transcription via transcription factors Swi4, Swi6, and Rlm1 (Levin, 2005). In *N. crassa*, the MAK-1 protein localizes to both the cytoplasm and the nucleus, indicating that it may also play a role in regulating transcription (Dettmann et al., 2013). The circadian rhythmic pattern of expression for some *mak-1*-dependent genes mirrors the rhythmic expression pattern of genes dependent on the transcription factor ADV-1; both *mak-1* and *adv-1* are targets of the circadian clock (Bennett et al., 2013; Dekhang et al., 2017). Furthermore, MAK-1 is required for transcription of *adv-1*, and misexpression of *adv-1* is sufficient to suppress the distinct colony morphology, slow growth rate, and short aerial hyphae phenotypes of the *Δmak-1* mutant (Fischer et al., 2018).

## The MAK-2 Signal Response Pathway

The core components of the MAK-2 signal transduction pathway are NRC-1 (MAPKKK), MEK-2 (MAPKK) and MAK-2 (MAPK). These MAP kinases are orthologous to the pheromone response pathway in *S. cerevisiae*, and the ERK1/2 pathway in mammals, which regulates diverse processes such as cell movement, differentiation, proliferation, and apoptosis (Shaul and Seger, 2007; Mendoza et al., 2015). HAM-5 has been identified as a scaffold for the MAK-2 pathway in *N. crassa* (Dettmann et al., 2014; Jonkers et al., 2014), which is not orthologous to Ste5p, the scaffold protein for the pheromone response MAPK pathway in *S. cerevisiae* (Printen and Sprague, 1994; Malleshaiah et al., 2010). The *ham-5* gene is restricted to the filamentous Ascomycetes (Pezizomycotina) and is required for communication, fusion, growth, perithecial development, and function of the MAK-2 cascade (Jamet-Vierny et al., 2007; Aldabbous et al., 2010; Dettmann et al., 2014; Jonkers et al., 2014).

In germlings and hyphae undergoing chemotropic interactions, the HAM-5, MAK-2, MEK-2, NRC-1, and STE-50 proteins form a complex that dynamically assembles and disassembles at roughly 4 min intervals at germ tube tips or hyphal fusion tips, mirroring the dynamic oscillations of SOFT (Fleissner et al., 2009; Dettmann et al., 2012; Jonkers et al., 2014). The MAK-2 complex assembles at the plasma membrane at germ tube tips, and when it disassembles, the components of the complex become diffuse in the cytoplasm (Fleissner et al., 2009). When the MAK-2 complex is assembled at the tip of one germ tube, at the tip of the opposing germ tube is a complex containing the CWI pathway scaffold protein, SOFT (Herzog et al., 2015; Fleißner and Herzog, 2016). Dynamics of SOFT-complex assembly and disassembly is equivalent to, but perfectly out of phase with MAK-2-complex dynamics at germ tube tips and hyphal fusion tips (Fleissner et al., 2009). Oscillation of MAK-2 to germ tube tips is required for chemotropic interactions, as evidenced by tethering MAK-2 to the plasma membrane, which disrupts communication (Serrano et al., 2018). The observation of MAK-2 and SOFT dynamic oscillations led to a “ping-pong” model (Goryachev et al., 2012), in which SOFT is predicted to be involved in signal secretion, while the MAK-2 pathway is predicted to be involved in signal reception. By temporally separating signal sending and signal receiving, genetically identical germlings could to avoid self-stimulation and maintain communication and chemotropic growth toward a physiologically distinct, but genetically identical clone.

The upstream regulators of the MAK-2 pathway in *N. crassa* are largely unknown, except for STE-50 (a conserved kinase regulator), STE-20 (PAK kinase), and RAS-2 (GTPase). In *N. crassa*, these three proteins physically interact with members of the MAK-2 complex and are required for phosphorylation of MAK-2 (Dettmann et al., 2014). Additionally, the conserved Rho GTPases CDC-42 and RAC-1 are necessary for maintaining polarized growth and cell fusion in *N. crassa*; when RAC-1 was inhibited, oscillatory recruitment of MAK-2 was stopped (Lichius et al., 2014). In mammals, activation of the ERK1/2 pathway generally begins with signal reception via a tyrosine receptor kinase, which activates a Ras GTPase, which then activates the ERK1/2 cascade. Ccd42, PAK Kinases (STE-20-like), and Rac GTPases also often act to modulate ERK1/2 signaling (Shaul and Seger, 2007). In *S. cerevisiae*, activation of the pheromone response pathway begins with signal reception via a G-Protein Coupled Receptor (GPCR; Ste2p or Ste3p), which leads to disassociation of the G-proteins, which activates Ste20p, Ste50p, and the Far1p/Cdc24p complex, and ultimately activates the Fus3p (*mak-2* ortholog) MAPK cascade (Bardwell, 2005). Tyrosine receptor kinases are conserved in the Opisthokonts and Amoebozoa, but were lost in the Ascomycota (including *N. crassa*), Ustilaginomycotina, and Pucciniomycotina lineages (Zhao et al., 2014). Thus, based on homology to the *S. cerevisiae* pheromone response pathway, it is expected that a GPCR activates the MAK-2 pathway in *N. crassa.* However, the *N. crassa* pheromone GPCR receptors and G-proteins are completely dispensable for somatic communication and cell fusion, although they play an important role during mating cell fusion and sexual development (Kim and Borkovich, 2004, 2006).

One intriguing component of the MAK-2 cascade in *N. crassa* is the NDR-kinase scaffold, HYM-1. NDR-kinases such as COT-1 regulate actin dynamics that define cell polarity (Yarden et al., 1992; Ziv et al., 2009), but are generally not essential for communication or fusion in *N. crassa*. HYM-1 is the scaffold protein for the COT-1 pathway, but HYM-1 also physically interacts the STE-20, and is required for the sequential phosphorylation of NRC-1, MEK-2, and MAK-2 (Dettmann et al., 2012). This observation suggests that HYM-1 is the bridge that connects MAK-2 pathway signaling with actin dynamics and cell polarity. However, the MAK-2 complex does not co-localize with actin markers, which indicates that the connection between MAK-2 and actin dynamics may be transient or indirect (Jonkers et al., 2016). In communicating germlings, both HYM-1 and COT-1 localize to the germ tube tip and do not oscillate dynamically, like MAK-2 or SOFT (Dettmann et al., 2012).

The transcription factor PP-1 (ortholog of Ste12p) is a conserved downstream target of MAK-2, and the *Δpp-1* mutant phenocopies the *Δmak-2* mutant (Li, 2005). A microarray expression study demonstrated that there is some overlap between MAK-2-dependent and PP-1-dependent gene expression (Leeder et al., 2013). However misexpression of *pp-1* in a *Δmak-2* background is not sufficient to induce transcription of downstream genes, which suggests that MAK-2 may directly activate, or de-repress PP-1 (Fischer et al., 2018). Consistent with this hypothesis is the observation that the PP-1 protein has several phosphorylation sites that are phosphorylated in a MAK-2-dependent manner (Jonkers et al., 2016).

## The Stripak Complex

The Striatin-Interacting protein Phosphatase And Kinase (STRIPAK) complex is a highly conserved multi-subunit protein complex that regulates diverse processes such as cytoskeletal organization, cell migration, and cell morphology in mammalian cells (Goudreault et al., 2009). In fungal cells, the STRIPAK complex is important for sexual development, growth, and cell fusion (Simonin et al., 2010; Dettmann et al., 2013; Kück et al., 2016). As evidence of its broad evolutionary conservation, the phenotype of the *S. macrospora* Striatin mutant was complemented with the mouse Striatin gene (Pöggeler and Kück, 2004). The STRIPAK complex in *N. crassa* consists of six proteins; PPG-1 (serine/threonine phosphatase), PP2A-A (phosphatase scaffold), HAM-3 (Striatin, phosphatase regulator), HAM-2 (membrane anchor), HAM-4 (membrane anchor), and MOB-3 (kinase activator) (Simonin et al., 2010; Dettmann et al., 2013). PP2A-A, HAM-3, and PPG-1 are the core members of a highly conserved phosphatase complex called PP2A that can function alone or in association with other complexes like the STRIPAK complex (Kabi and McDonald, 2014). The PP2A complex is considered to be the major kinase phosphatase in eukaryotic cells, and defines a central signaling hub for many eukaryotic signaling pathways. No kinases have been identified as components of the STRIPAK complex in *N. crassa*, however, in closely related *S. macrospora*, two kinases (KIN3 and KIN24) were shown to have a weak physical interaction with the Striatin ortholog in the STRIPAK complex (Frey et al., 2015); KIN3 is required for fruiting body formation, hyphal fusion, and septation (Radchenko et al., 2018). The homolog of KIN24 in *N. crassa* is MST-1 (NCU00772), which physically interacts with NRC-1 and MEK-2 (Dettmann et al., 2014), indicating that MST-1 may physically connect the MAK-2 pathway with the STRIPAK complex. However, the *mst-1* gene is dispensable for germling communication and cell fusion. The MST-1 and the KIN3-ortholog STK-3 localize to septa and spindle pole-bodies, and were thus hypothesized to regulate septation and polarized growth in *N. crassa* (Frey et al., 2015; Heilig et al., 2014). Lastly, a SIKE-like small coiled-coil protein, SCI1, was recently identified as a component of the STRIPAK complex in *S. macrospora* (Reschka et al., 2018). The SCI1 protein physically interacts with the orthologs of HAM-3 and HAM-4, and the *Δsci1* mutant displays defects in hyphal fusion, perithecial development, and growth rate (Reschka et al., 2018).

## Crosstalk Between the CWI Pathway, the MAK-2 Pathway, and the Stripak Complex

**FIGURE 2:**
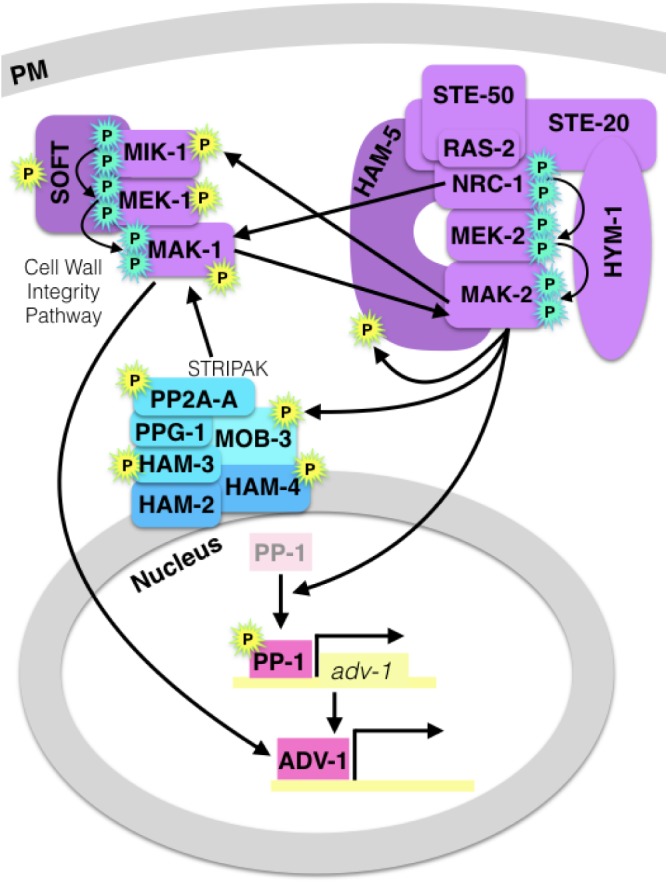
Diagram of crosstalk between the MAK-2 pathway, the CWI/MAK-1 pathway and the STRIPAK complex. A cyan “P” indicates canonical MAPK cascade phosphorylation, and a yellow “P” indicates MAK-2-dependent phosphorylation. MAPK pathway proteins are purple, the STRIPAK complex is blue, and pink transcription factors (PP-1 and ADV-1) are downstream of all three signaling components. MAK-2 is required for activation or de-repression of PP-1, and MAK-1 regulates ADV-1 in a PP-1-independent manner.

There are several points of crosstalk between the CWI pathway, the MAK-2 pathway, and the STRIPAK complex, which is summarized in Figure 2. Both the MAK-1 and the MAK-2 pathways regulate gene transcription via the transcription factors PP-1 and ADV-1 (Leeder et al., 2013; Dekhang et al., 2017; Fischer et al., 2018). Briefly, MAK-2 is required for activation or de-repression of PP-1, then PP-1 directly activates transcription of ADV-1, and ADV-1 directly activates transcription of many genes that are required for communication, fusion, growth, and cell wall stress response, including *mek-1*. Additionally, MAK-1 was shown to function upstream of ADV-1, independently of PP-1 (Fischer et al., 2018). The *ham-7, mik-1, mek-1*, and *nrc-1* genes are all required for full phosphorylation of MAK-1 (Maddi et al., 2012; Leeder et al., 2013; Fu et al., 2014; Teichert et al., 2014) and both *ham-7* and *mak-1* have been shown to be required for full phosphorylation of MAK-2 (Maerz et al., 2008; Dettmann et al., 2012; Maddi et al., 2012; Leeder et al., 2013). These data suggest that HAM-7 either functions upstream of the MAK-1 cascade and influences MAK-2 phosphorylation indirectly via MAK-1, or HAM-7 functions directly upstream of both the CWI and MAK-2 pathways.

MAP kinases post-translationally regulate other proteins via phosphorylation, and MAK-2 is required for the phosphorylation of several proteins that are important for communication and cell fusion (Table 1). Catalytically active MAK-2 is required for phosphorylation of kinases and scaffold proteins in both the MAK-2 and CWI pathways (Jonkers et al., 2014). MAK-2 also phosphorylates MOB-3, which affects accumulation of MAK-1 in the nucleus (Dettmann et al., 2013). Both *mob-3* and *ham-3* are required for full phosphorylation of MAK-1. In addition to MOB-3, three other STRIPAK proteins; HAM-3, HAM-4, and PP2A-A are also phosphorylated in a MAK-2-dependent manner.

**Table 1:** Communication proteins that are phosphorylated in a MAK-2-dependent manner.

Gene ID	Annotation	Gene Name	Number of phosphorylated residues	Pathway or Function
NCU02234	MAP kinase kinase kinase	*mik-1*	1	CWI pathway
NCU06419	MAP kinase kinase	*mek-1*	3	CWI pathway
NCU09842	MAP kinase	*mak-1*	2	CWI pathway
NCU02794	Scaffold protein	*so*	16	CWI pathway
NCU06182	MAP kinase kinase kinase	*nrc-1*	1	MAK-2 pathway
NCU04612	MAP kinase kinase	*mek-2*	3	MAK-2 pathway
NCU01789	Scaffold protein	*ham-5*	3	MAK-2 pathway
NCU00340	Transcription factor STE12	*pp-1*	7	MAK-2 pathway
NCU00488	Protein phosphatase PP2A regulatory subunit	*pp2A-A*	1	STRIPAK
NCU00528	Hyphal anastamosis protein 4	*ham-4*	1	STRIPAK
NCU08741	Hyphal anastamosis protein 3	*ham-3*	2	STRIPAK
NCU05364	Tyrosine-protein phosphatase	*ptp-2*	4	phosphatase
NCU08380	Plasma membrane phosphatase	*csp-6*	2	phosphatase
NCU01833	Two-component histidine kinase CHK-1	*nik-2*	4	kinase
NCU07280	Serine/threonine-protein kinase gad8	*ypk-1*	2	kinase
NCU05485	Casein kinase II regulatory subunit	*ckb-1*	3	kinase regulator
NCU06067	Rho guanyl nucleotide exchange factor	*cdc-24*	6	GEF
NCU06500	Cell division control protein 25	*cdc-25*	1	GEF
NCU07850	NADPH oxidase regulator NoxR	*nor-1*	3	ROS
NCU07192	Determinant of communication 2	*doc-2*	11	non-self recognition
NCU02811	Hyphal anastamosis-8 protein	*ham-8*	2	membrane protein
NCU07389	Hyphal anastamosis protein 9	*ham-9*	7	membrane protein
NCU04732	Hyphal anastamosis-11 protein	*ham-11*	6	membrane protein
NCU06265	Hyphal anastamosis-13 protein	*ham-13*	1	unknown
NCU07238	Hyphal anastamosis-14 protein	*ham-14*	9	unknown
NCU05622	Beta-Ala-His dipeptidase	*arg-15*	1	unknown
NCU08377	Adenylate cyclase	*cr-1*	5	Actin dynamics
NCU04645	DUF124 domain-containing protein	n/a	1	unknown


*Adapted from Jonkers et al. (2014)*.

Additional evidence for STRIPAK-mediated crosstalk between the CWI and MAK-2 pathways was demonstrated via protein-protein interaction experiments. An Affinity Purification-Mass Spectrometry (AP-MS) experiment identified a weak interaction between MAK-2 and the STRIPAK components PPG-1, PP2A-A, HAM-3, and MOB-3 (Dettmann et al., 2014). Furthermore, MST-1 was identified by both AP-MS and Y2H as interacting with NRC-1 and MEK-2 and, in *S. macrospora*, the ortholog of MST-1 was found to have a weak interaction with the HAM-3-homolog (Dettmann et al., 2014; Frey et al., 2015).

## Phosphatases

The magnitude and duration of phosphorylation determines the signaling output of a kinase pathway, and phosphatases play a critical role in modulating protein phosphorylation (Behar et al., 2008; Caunt and Keyse, 2013). For example, constitutive activation of the Ras protein causes *Schizosaccharomyces pombe* cells to initiate fusion prior to making contact with a fusion partner (Merlini et al., 2018). The phosphatase genes *csp-6* and *ppg-1* are required for germling communication and cell fusion in *N. crassa* (Dettmann et al., 2013) and the *ptp-2* gene is the ortholog of the phosphatase that negatively regulates the *mak-2*-ortholog in *S. cerevisiae*. The *Δptp-2* mutant is not defective at germling communication or fusion, but the PTP-2 protein is phosphorylated in a MAK-2-dependent manner, which may be indicative of a feedback loop in which MAK-2 regulates itself via PTP-2 (Jonkers et al., 2016). MAK-2 also regulates phosphorylation of CSP-6 and PP2A-A, which is the PPG-1 (phosphatase) regulatory subunit of the STRIPAK complex.

The *csp-6* gene was initially identified in *N. crassa* in a screen of phosphatase mutants (Ghosh et al., 2014). The *Δcsp-6* mutant has a pleiotropic phenotype that is defective at conidial separation, growth rate, female sexual development, and circadian clock regulated developmental oscillations, and is sensitive to osmotic stress (Chinnici et al., 2014; Ghosh et al., 2014; Zhou et al., 2018). The CSP-6 protein is orthologous to Psr1p and Psr2p in *S. cerevisiae*, which physically interact with Whi2p to mediate the general stress response pathway (Kaida et al., 2002). Similarly, the CSP-6 protein physically interacts with the WHI-2 protein in *N. crassa* (Zhou et al., 2018). The CSP-6 protein was shown to dephosphorylate the main circadian clock regulator, WC-1, which affects ADV-1-dependent transcription. Furthermore, both CSP-6 and WHI-2 localize to the cytoplasm and the nucleus (Zhou et al., 2018). While phosphorylation of MAK-1 and MAK-2 in a *Δcsp-6* background has not been assessed, phosphorylation of both MAK-1 and MAK-2 is dramatically reduced in the *Δwhi-2* mutant (Fu et al., 2014), indicating that WHI-2, and likely CSP-6, function upstream of MAK-1 and MAK-2. Together with the STRIPAK complex, CSP-6 and WHI-2 may function as a signaling hub that contributes to the integration of multiple signaling pathways.

## Transcription Factors

Two transcription factors, *adv-1* and *pp-1* are necessary for germling communication and fusion in *N. crassa* (Leeder et al., 2013; Dekhang et al., 2017). PP-1 is a C2H2-Zn^2+^ transcription factor that is homologous to the pheromone response pathway transcription factor, Ste12p in *S. cerevisiae* (Leeder et al., 2013). In *S. cerevisiae*, the pheromone response pathway is orthologous to the MAK-2 pathway and results in de-repression of the Ste12p transcription factor (Bardwell, 2005; Merlini et al., 2013). Ste12-like proteins, including PP-1, have two C2H2-Zn^2+^ motifs and a homeobox-like STE domain that is involved in binding DNA (Errede and Ammerer, 1989). In *N. crassa* the STE domain is essential for function, but the C2H2-Zn^2+^ motifs are dispensable (Leeder et al., 2013). Ste12-like transcription factors are regulated by direct phosphorylation and phosphorylation of associated regulatory proteins (Blackwell et al., 2007). Several phosphorylation sites have been identified on PP-1 in *N. crassa*, but the biological significance of these sites remains unknown (Leeder et al., 2013; Jonkers et al., 2014; Xiong et al., 2014). Additionally, there are no known regulatory proteins that bind PP-1 in a way that is analogous to how Dig1p/Dig2p bind and repress Ste12p in *S. cerevisiae* (McCullagh et al., 2010). However, it has been suggested that a nuclear protein called NsiA may physically bind and positively regulate the ortholog of *pp-1* in the plant endophyte, *Epichloë festucae* (Green, 2016).

Ste12-like proteins have been well studied and they regulate diverse functions throughout the fungal kingdom, such as growth, virulence, sex, dimorphism, and asexual development (Hoi and Dumas, 2010). Ste12p and its homologs in the yeasts *S. cerevisiae* and *S. pombe* are essential for sexual reproduction, as they respond to pheromone stimulus and activate transcription of mating-specific genes (Bardwell, 2005; Merlini et al., 2013). Ste12p also regulates the morphological switch from yeast to pseudohyphal growth in response to starvation (Roberts and Fink, 1994). In addition to regulating sexual reproduction and morphological switching, a Ste12 homolog also regulates virulence in the animal-pathogenic yeasts *Candida glabrata* and *Cryptococcus gattii* (Calcagno et al., 2003; Ren P. et al., 2006). In the dimorphic opportunistic human pathogen *Penicillium marneffei*, the Ste12-homolog StlA is dispensable for the switch from the yeast to hyphal morphology and has no other known function. However, the identification of StlA in the genome suggests that cryptic sex exists in *P. marneffei*; *stlA* from *P. marneffei* is sufficient to complement the sexual defects in the *Aspergillus nidulans* mutant, *ΔsteA* (Borneman et al., 2001). In *A. nidulans*, SteA is necessary for sexual reproduction, but dispensable for asexual growth and development (Vallim et al., 2000). In contrast, other filamentous fungi depend on Ste12 to regulate growth, asexual development, and pathogenicity. The Ste12 homolog in *N. crassa* and *S. macrospora* is not essential for mating cell fusion, but it is essential for wild-type-like growth, somatic cell fusion, protoperithecium development (*N. crassa* only), ascus development, and ascospore germination (Colot et al., 2006; Nolting and Pöggeler, 2006; Leeder et al., 2013). In *S. macrospora*, the NADPH-oxidase *nox2* is specifically required for ascospore development, and there is overlap in the differentially expressed genes in the *Δnox2* and *Δste12* mutants as compared to wild-type cells (Dirschnabel et al., 2014). Filamentous plant pathogenic fungi (i.e., *Magnaporthe oryzae*, *Alternaria brassicola, Verticillium dahlia, Fusarium oxysporum*, and *Colletotrichum lagenarium*) require the Ste12-homolog for either appressorium formation or plant penetration, and subsequent pathogenesis on the plant host. Furthermore, the Ste12-homolog in these plant pathogens is dispensable for sexual reproduction where the sexual cycle is observable (Park et al., 2002; Tsuji et al., 2003; Cho et al., 2009; Rispail and Di Pietro, 2010; Sarmiento-Villamil et al., 2018). In contrast, the plant pathogen *Cryphonectria parasitica* requires a Ste12-homolog to regulate both female fertility and virulence (Sun et al., 2009).

ADV-1 is a Zn(II)_2_Cys_6_ transcription factor that, like PP-1, regulates growth, sex, virulence, sexual development and cell fusion (Colot et al., 2006; Dekhang et al., 2017). ADV-1 regulated transcription is a target of the CWI and MAK-2 pathways (Fischer et al., 2018). In contrast to PP-1, ADV-1 has also been shown to be a target of the circadian clock master regulator, the White Collar Complex (WCC) (Dekhang et al., 2017). ADV-1 is essential for developmental oscillations (i.e., conidiation) and the quantity of *adv-1* mRNA and protein correlates well with the circadian clock, though *adv-1* expression is never fully off (Smith et al., 2010). The expression of ADV-1 target genes in a mature colony also occurs rhythmically and in concert with the circadian clock. These data led to the hypothesis that hyphal fusion within a fungal colony is a clock-regulated developmental process (Dekhang et al., 2017).

ADV-1 is not as broadly conserved as PP-1, as clear *adv-1* homologs are restricted to the filamentous Ascomycetes (Pezizomycotina). In *Aspergillus* spp., the *adv-1* homolog RosA represses fruiting body development whereas a second *adv-1* homolog, NosA, induces fruiting body development. *nosA* and *rosA* share 43% sequence similarity, and *nosA* is a target of the metabolism and the developmental regulator, LaeA (Vienken et al., 2005; Vienken and Fischer, 2006; Zhao et al., 2017). In *S. macrospora*, the *pro1* gene is the homolog of *adv-1*, and the *N. crassa adv-1* gene is sufficient to complement the *S. macrospora Δpro1* mutant. A *pro1*-like gene (named *hpro1A*) was identified in both *N. crassa* and *S. macrospora* that shares 40% similarity with *pro1* and *adv-1*, however, this potential paralog does not complement the function a *S. macrospora Δpro1* mutant (Masloff et al., 2002). It remains unclear if *hpro1A* plays a role in communication, fusion, or related processes in either species. PRO1, like other Zn(II)_2_Cys_6_ transcription factors, has a Gal4-like DNA-binding domain and a transcription-activation domain. However, unlike Gal4p, PRO1 lacks a coiled-coil dimerization domain, indicating that PRO1 likely works alone (Masloff et al., 2002). The *S. macrospora Δpro1* mutant and the *N. crassa Δadv-1* mutant both produce protoperithecia, but are defective in the early stages of perithecial development (Masloff et al., 1999; Fischer et al., 2018). In plant-pathogenic *A. brassicicola*, the *adv-1* homolog is required for growth and may also be involved in virulence (Cho et al., 2009). Another plant pathogen, *C. parasitica*, does not require the *adv-1* homolog for virulence, but does require it for female fertility and maintaining stable mycovirus infection (Sun et al., 2009). The *adv-1* homolog in the plant endophyte *E. festucae* is required for hyphal fusion and maintenance of mutualism. *E. festucae* is normally a mutualistic grass endophyte, but certain environmental conditions or the deletion of specific genes, such as the *adv-1* homolog, cause *E. festucae* to switch to a pathogenic lifestyle, resulting in stunted growth of the host plant (Tanaka et al., 2013).

## The NOX Complex and Reactive Oxygen Species

While Reactive Oxygen Species (ROS) can be destructive, ROS are also known to be an important aspect of several eukaryotic signaling pathways including cell differentiation, development, and cytoskeletal remodeling ( Hansberg et al., 1993; Finkel, 2003; Aguirre et al., 2005). NADPH-oxidases (NOX) are evolutionarily conserved and produce superoxide by oxidizing NADPH and reducing molecular oxygen. Three different *nox* gene subfamilies have been identified in *Aspergillus* spp.: *noxA*, *noxB*, and *noxC* (Aguirre et al., 2005). The genome of *N. crassa* has *nox-1* (*noxA*) and *nox-2* (*noxB*) homologs, but not a *noxC* homolog (Galagan et al., 2003). The *noxA* and *noxB* homologs have diverse functions in fungi, but in general, *noxA* is important for a variety of processes including fruiting body development, cell fusion, growth, sclerotial development, and virulence of plant pathogens (Lara-Ortíz et al., 2003; Malagnac et al., 2004; Egan et al., 2007; Cano-Dominguez et al., 2008; Segmüller et al., 2008; Dirschnabel et al., 2014). The *noxB* homologs often have a more specific function such as ascospore development or mediating appressorium penetration of a host plant (Egan et al., 2007; Cano-Dominguez et al., 2008; Segmüller et al., 2008; Tanaka et al., 2008; Dirschnabel et al., 2014). In *S. macrospora*, many of the genes required for communication, cell fusion, and sexual development are differentially expressed in a *Δnox-1* mutant as compared to wild-type hyphae/protoperithecia (Dirschnabel et al., 2014). These data suggest that the NOX-1 complex likely functions upstream of a transcriptional response during communication and cell fusion.

The *nor-1* gene is a conserved regulatory subunit of both *nox-1* and *nox-2*, however, only *nox-1* and *nor-1* are required for germling communication, cell fusion, growth, and female sexual development in *N. crassa* (Cano-Dominguez et al., 2008). NoxR (*nor-1* ortholog) was first described in the plant endophyte *E. festucae*, and was shown to activate Nox1 in a complex with the conserved regulatory proteins, BemA, Cdc24, and RacA (Takemoto et al., 2006, 2011; Tanaka et al., 2006, 2008). Recently, Cdc42 was identified as a negative regulator of Nox1 in *E. festucae*, and which also physically interacts with BemA (Kayano et al., 2018). Orthologs of BemA, RacA, Cdc24, and Cdc42 are conserved general regulators of polarized growth and cytoskeletal dynamics in fungi and have been particularly well characterized in *S. cerevisiae* (Drees et al., 2001; Martin and Arkowitz, 2014). Strains containing mutations in orthologs of RacA, Cdc24, and Cdc42 in *N. crassa* and other filamentous fungi have a strong pleiotropic phenotype, while strains carrying mutations in the BemA ortholog are less pleiotropic and are not severely compromised for polarized growth (Araujo-Palomares et al., 2011; Takemoto et al., 2011; Schürg et al., 2012). However, these four proteins may represent a link between cytoskeletal dynamics and the NOX complex.

The HAM-6 protein is a component of the NOX-1 complex in filamentous fungi. The *ham-6* gene was initially identified in *S. macrospora* and *N. crassa* in screens for mutants defective at sexual development and germling fusion (Nowrousian et al., 2007; Fu et al., 2011). This gene encodes a transmembrane domain protein that is conserved among the filamentous Ascomycetes (Pezizomycotina). Recent research demonstrated that HAM-6 is a necessary component of the NOX-1 complex in filamentous fungi. The *ham-6* ortholog in *Botrytis cinerea* and *Podospora anserina* (a close relative of *N. crassa*) physically and genetically interacts with the *nox-1* and *nor-1* orthologs (Lacaze et al., 2015; Siegmund et al., 2015; Marschall et al., 2016). The *ham-6* gene is differentially expressed in *Δnox-1* cells compared to wild-type, and *ham-6* is epispastic to *soft* in *S. macrospora* (Nowrousian et al., 2007; Dirschnabel et al., 2014). Interestingly, *ham-6* is required for full phosphorylation of MAK-1 (Fu et al., 2014), indicating that HAM-6, and potentially the NOX-1 complex, function upstream of the CWI pathway. Additional evidence of a connection between the NOX-1 complex and the CWI pathway was demonstrated in the mycoparasitic Dothidiomycete, *Coniothyrium minitans*, in which the MAK-1 ortholog physically interacts with the NOX-1 ortholog (Wei et al., 2016). Furthermore, over-expression of the *mak-1*-ortholog was sufficient to partially suppress the phenotype of the *Δrac-1* mutant (Wei et al., 2016). In *B. cinerea*, Nox1 was also recently shown to physically interact with Mak1 via the scaffold protein Iqg1 (Marschall and Tudzynski, 2016). The Iqg1 protein is a conserved scaffold that links multiple signaling pathways, including the NOX and CWI pathways (Marschall and Tudzynski, 2016).

The superoxide produced by NOX enzymes is highly reactive, short-lived, and unable to cross biological membranes because of the negative charge. It is therefore highly unlikely that ROS functions as a signal mediating germling communication. However, ROS could play an indirect roll in extracellular signaling. Recent evidence in *B. cinerea* indicates that ER localization and the N-terminus of NOX-1 are both essential for mediating plant pathogenicity, while plasma membrane localization and the C-terminus of NOX-1 are essential for mediating germling communication and fusion (Siegmund et al., 2015; Marschall et al., 2016). These data indicate that the NOX-1 complex regulates germling communication and fusion from the plasma membrane. However, it is unclear if ROS are directly important for signaling, or if the NOX-1 complex is primarily important for coordinating cytoskeletal dynamics with other signaling pathways.

## Cytoskeleton and Secretion

The actin cytoskeleton in fungi is important for mediating cell polarity, septation, exocytosis, endocytosis, organelle movement, and chemotropic growth (Huckaba et al., 2004; Moseley and Goode, 2006; Roca et al., 2010; Berepiki et al., 2010). In *N. crassa* germlings undergoing chemotropic interactions, actin cables and actin patches are dramatically enriched at CAT tips (Roca et al., 2010). Actin patches are associated with endocytic vesicles, while actin cables are associated with exocytosis and sites of growth and organelle movement. When two communicating germlings make contact and begin the process of cell fusion, the actin cables slowly disappear, while the patches remain throughout the fusion process (Berepiki et al., 2010; Roca et al., 2010). When germlings were treated with the microtubule-inhibitor Benomyl, no effect on communication or cell fusion was observed. In contrast, when germlings were treated with a sub-lethal dose of the actin-polymerization-inhibitor Lactrunculin B, germling communication and fusion was significantly reduced (Roca et al., 2010). Together, these data demonstrate that microtubules are dispensable for germling communication, while actin is critically important.

The Arp2/3 complex is a highly conserved actin-regulatory complex in all Eukaryotes (Robinson et al., 2001). This complex has been well characterized in vertebrates, *S. cerevisiae*, and *Arabidopsis thaliana*, but is largely uninvestigated in filamentous fungi. The Arp2/3 complex consists of seven subunits, the names of which are not consistent between organisms (Table 2). These genes are generally considered to be essential in *N. crassa*, however, mutants of *arp-6*, *arp-14*, and *arp-13* were shown to be defective at germling communication and fusion (Roca et al., 2010). Both ARP-2 and ARP-3 co-localize with actin in *N. crassa* and these proteins were developed as actin reporters (Delgado-Álvarez et al., 2010).

**Table 2:** Arp2/3-complex genes in *N. crassa* and other organisms.

*N. crassa* Gene ID	*N. crassa* Gene Name	*S. cerevisiae* Gene Name	*A. thaliana* and Human Gene Name	Common Vertebrate Gene Name	Function
NCU07171	*arp-6/arp-2*	Arp2	Arp2	Arp2	Similar to monomeric actin
NCU01756	*arp-3*	Arp3	Arp3	Arp3	Similar to monomeric actin
NCU02781	*–*	Arc40/Sop2	ARPC1	p41	WD40 β-propeller protein
NCU03050	*arp-15*	Arc35	ARPC2	p34	Structural backbone subunit
NCU09572	*arp-14*	Arc18	ARPC3	p21	Tether for Arp3
NCU01918	*arp-13*	Arc19	ARPC4	p19	Structural backbone subunit
NCU03438	*arp-12*	Arc15	ARPC5	p16	Tether for Arp2


*Adapted from Pizarro-Cerdá et al. (2017)*.

Proteins that are peripherally associated with the MAK-2 pathway in *N. crassa* are involved in regulating actin dynamics. The HYM-1 protein is the scaffold protein for COT-1 and POD-6, which are involved in regulating actin dynamics and polarized growth in both fungi and animals (Baas et al., 2004; Maerz et al., 2008). The HYM-1 protein is required for the sequential phosphorylation of the MAK-2 MAPK cascade, and HYM-1 and STE-20 physically interact in *N. crassa* (Dettmann et al., 2012). The *ste-20* gene encodes a conserved upstream Pak1-like kinase that connects multiple signaling pathways that are involved in polarized growth and signal response. In *S. cerevisiae*, Bem1p interacts with both Ste20p and actin to regulate polarized growth (Leeuw et al., 1995). In mammalian cells, the *ste-20*-ortholog phosphorylates members of the Arp2/3-complex to directly regulate actin dynamics (Vadlamudi et al., 2004). An adenylate cyclase (CR-1) and its associated adenylate cyclase capping protein (CAP-1) physically interact with the MAK-2 pathway in *N. crassa* via the conserved MAP-kinase regulator, STE-50 (Dettmann et al., 2014). The CAP-1/CR-1 complex binds actin monomers, is regulated by RAS-2, and has been shown to be involved in regulating actin dynamics and cell polarity in diverse fungi (Rocha et al., 2001; Bertling et al., 2007; Zhou et al., 2012). Lastly, a myosin mutant, *Δmyo-2* was shown to be defective at germling communication and fusion in *N. crassa* (Dettmann et al., 2014). These data combined demonstrate the central role of actin and its associated regulatory proteins during germling communication, which involves polarized growth.

Actin is important for mediating endocytosis, exocytosis, and vesicle tracking, which are all necessary for polarized growth and fusion. Amphiphysins regulate membrane curvature, vesicle trafficking, endocytosis, exocytosis, and actin organization (Sivadon et al., 1995; Ren G. et al., 2006; Toume and Tani, 2016). The *amph-1* gene in *N. crassa* encodes an amphiphysin-like protein with a Bin-Amphiphysin-Rvs (BAR) domain; an *Δamph-1* mutant in *N. crassa* is defective at germling communication, cell fusion, growth, and sexual development (Fu et al., 2011, 2014). Proteins containing a BAR domain are involved in protein-protein interactions and create a scaffold for regulating membrane curvature and connecting membrane dynamics with the cytoskeleton (Sivadon et al., 1995; Ren G. et al., 2006; Toume and Tani, 2016). The *amph-1* homologs in *S. cerevisiae* are *RVS161* and *RVS167*, which are required for polarization of the actin cytoskeleton, polarized growth, cell fusion, and endocytosis (Youn et al., 2010). Amphiphysins regulate exocytosis in neuronal synaptic cells in *Drosophila melanogaster* (Mathew et al., 2003) and have been well studied in animals where these proteins interact with actin-regulatory proteins (i.e., Arp2/3) and Rho GTPases (i.e., Cdc42) to mediate endo- and exocytosis of synaptic vesicles (Aspenström, 2014).

A genome wide association study of *N. crassa* wild isolates identified several additional genes that are required for communication and are involved in the secretory pathway, endocytosis, or exocytosis (Palma-Guerrero et al., 2013). The GTPase activating protein Gyp5p physically interacts with amphyphysins to recruit them to sites of polarized growth and endocytosis or exocytosis in *S. cerevisiae* (Prigent et al., 2011). The *Δgyp-5* mutant in *N. crassa* is a hyper-fusion mutant that fuses significantly more than wild-type cells (Palma-Guerrero et al., 2013). The Sec15 gene encodes an essential member of the exocyst complex in *S. cerevisiae*, and the *Δsec-15* mutant in *N. crassa* is defective at germling communication (Palma-Guerrero et al., 2013). The *sec-22* gene encodes a SNARE protein that is also involved in vesicle trafficking, particularly between the ER and Golgi (Liu and Barlowe, 2002). The *Δsec-22* mutant in *N. crassa* has a reduced frequency of germling communication (Palma-Guerrero et al., 2013), and in *S. macrospora, sec22* is necessary for the later stages of sexual development (Traeger and Nowrousian, 2015). In mammalian cells, a biochemical link has been established between the exocyst complex (including *sec-15*), GTPase activating proteins (i.e., *gyp-5*), Rac1, and BAR-domain containing proteins (i.e., *amph-1*), which all work together to regulate cell motility (Parrini et al., 2011). It is possible that a similar regulatory network exists in fungi for mediating polarized growth, including chemotropic interactions.

## Calcium-Dependent Proteins

Calcium is important for mediating polarized hyphal growth, and extracellular calcium is necessary for germling communication in *N. crassa* (Palma-Guerrero et al., 2013; Takeshita et al., 2017). Furthermore, four different calmodulin-dependent kinases (CaMK) in *N. crassa* are required for growth, sexual development, and stress response (Kumar and Tamuli, 2014). The germling fusion phenotype of these CaMK mutants is unknown. Calcium plays a critical role during the process of membrane fusion, and some of the genes involved in communication are predicted either to bind calcium or function in a calcium-dependent manner. The HAM-3 (Striatin) protein in the STRIPAK complex has a binding motif specific for the calcium-signaling regulator, Calmodulin, and Striatins in general are well known to integrate with calcium signaling (Bartoli et al., 1998; Simonin et al., 2010). The *cch-1* gene encodes an integral membrane calcium channel, and the *Δcch-1* mutant has a reduced frequency of germling communication (Chu, 2013).

The *ham-10* gene is essential for germling communication and it encodes a putative C2-domain protein (Fu et al., 2011, 2014). The C2-domain exists across Eukarya and is defined by two 4-stranded β-sheets that bind phospholipids in a calcium-dependent manner. C2-domain proteins have diverse functions that are involved in various aspects of membrane repair, vesicle trafficking, anchoring cytoskeletal elements to membranes, and lipid-based signal transduction (Zhang and Aravind, 2010).

Homologs of three *N. crassa* genes (*cse-1*, *pik-1*, and *nfh-1*) are important for calcium-dependent exocytosis in mammals and *S. cerevisiae*. In *N. crassa*, the *Δcse-1*, *Δpik-1*, and *Δnfh-1* mutants all have a significantly reduced frequency of germling communication, and the CSE-1 protein co-localizes with Golgi markers (Palma-Guerrero et al., 2013). The CSE-1 protein is predicted to bind calcium ions, which directly impacts Calmodulin-dependent calcium signaling (Tamuli et al., 2011). In *S. cerevisiae*, the Pik1p physically interacts with Frq1p (CSE-1 homolog) and promotes its association with the Golgi, which is necessary for regulating calcium-dependent exocytosis. Additionally, in *S. cerevisiae* Pik1p is required for activation of the *mak-2*-ortholog, Fus3p (Cappell and Dohlman, 2011). The *S. cerevisiae nfh-1*-ortholog is a regulator that also binds Pik1p and shuttles it between the nucleus and cytoplasm (Demmel et al., 2008).

## Membrane Fusion

The result of cell-to-cell communication and chemotropic growth is cell fusion, and five genes have been identified that are specifically involved in membrane merger during cell fusion in *N. crassa*. An ergosterol mutant (*Δerg-2*) was recently identified that fails to arrest growth after two germlings make contact (Weichert et al., 2016). Thus, the sterol composition of the cell membrane may play an important role in mediating the switch from communication to cell fusion. The deletion mutants, *Δprm-1, Δlfd-1, Δlfd-2, Δfig-1*, and ΔNCU01697 engage in germling chemotropic growth, but are defective in the process of cell fusion and generally have increased levels of cell lysis, likely due to a compromised plasma membrane. The *prm-1* gene encodes a conserved integral membrane protein that localizes to the plasma membrane and is necessary for mediating membrane fusion after cell wall breakdown (Fleißner et al., 2008a). The *lfd-1* gene is unique to the filamentous Ascomycetes (Pezizomycotina) and encodes a protein with a single transmembrane domain that localizes to the plasma membrane. The phenotype of *Δlfd-1* mutant is remarkably similar to the *Δprm-1* mutant, and both mutants are abnormally sensitive to calcium-stress (Palma-Guerrero et al., 2014). The *Δlfd-2*, *Δfig-1*, and ΔNCU01697 mutants also have a calcium-dependent cell-lysis phenotype (Palma-Guerrero et al., 2015). The NCU01697 locus encodes an uncharacterized protein that is predicted to have an acyltransferase domain, which could be important for phospholipid biosynthesis. The LFD-2 protein resides in the membrane of the Golgi and ER, while the FIG-1 protein is a plasma membrane low-affinity calcium ion channel, and both proteins are important for regulating membrane homeostasis and membrane repair. Epistasis experiments demonstrated that LFD-2 and FIG-1 likely function in separate pathways (Palma-Guerrero et al., 2015).

## Non-Self Recognition Before and After Cell Fusion

Non-self recognition regulates chemotropic interactions between cells that have different allelic specificities at the *doc* loci (Heller et al., 2016). If cells have identical allelic specificity at the *doc* loci, robust chemotropic communication between cells ensues. However, if cells have different allelic specificities at the *doc* loci, chemotropic interactions are not reinforced and cell fusion frequencies are greatly reduced. Wild populations of *N. crassa* fall into five different communication groups that are correlated with five different *doc* haplotypes. Thus, the *doc* genes function as “greenbeard” genes (Heller et al., 2016), similar to genes in other organisms that regulate cooperation and cell identity. Greenbeard genes are involved in long-distance kind discrimination, leading to cooperation between non-genealogical relatives with similar greenbeard genes. Post-fusion non-self recognition mechanisms also exist in filamentous fungi whereby if two cells undergo communication and fusion, but carry different allelic specificities at heterokaryon incompatibility *(het)* loci, fusion cells undergo a type of programmed cell death (Saupe, 2000; Glass and Kaneko, 2003; Glass and Dementhon, 2006; Gonçalves et al., 2017). Some non-self recognition mechanisms mediated by genetic differences *at het* loci are only active in hyphae, because death is not immediately activated if two incompatible germlings undergo fusion (Ishikawa et al., 2012). In contrast, the incompatibility loci *sec-9* and *plp-1* mediate post-fusion programmed cell death between both germlings and in hyphae carrying incompatible *sec-9/plp-1* alleles (Heller et al., 2018).

## Miscellaneous and Hypothetical Communication Genes

Several genes have been identified as necessary for germling communication and fusion, but it is unclear how these genes fit into the regulatory network that mediates germling communication. Many of these loci were named “hyphal anastomosis mutant” (*ham*) genes, which reflected their uncharacterized nature. Below is a brief description of what is known about these genes.

### *ham* Genes

The *Δham-8* mutant is defective at communication, cell fusion, growth rate, and sexual development (Fu et al., 2011). The *ham-8* gene encodes a predicted transmembrane domain protein with four transmembrane domains characteristic of the mammalian MARVEL proteins, which regulate tight-junctions between mammalian cells and vesicle transport (Sánchez-Pulido et al., 2002). The *Δham-8* mutant in *N. crassa* also has reduced levels of phosphorylated MAK-1, and to a lesser extent, MAK-2 (Fu et al., 2014), which indicates that *ham-8* may function upstream of the CWI/MAK-1 pathway. The HAM-8 protein is phosphorylated in a MAK-2-dependent manner (Jonkers et al., 2014).

The *Δham-9* mutant is defective at communication, cell fusion, growth rate, and sexual development (Fu et al., 2011). The *ham-9* gene encodes a protein with a predicted pleckstrin domain, which transiently associates with lipid bilayers. Pleckstrin domains are common in a variety of proteins that are generally associated with signal transduction (Lemmon, 2007). The *Δham-9* mutant has wild-type-like levels of phosphorylated MAK-1 and MAK-2, indicating that the *ham-9* gene is not required for activation of these two MAPK pathways.

The *Δham-11* mutant has a unique phenotype that is unlike any other communication mutant (Leeder et al., 2013). The *Δham-11* mutant is indistinguishable from wild-type in terms of hyphal cell fusion, growth rate, aerial hyphae production, and sexual development, but *Δham-11* germlings fail to normally undergo chemotropic interactions or cell fusion. However, *Δham-11* germlings undergo chemotropic interactions and fusion with otherwise isogenic wild-type germlings (Leeder et al., 2013). Furthermore, SOFT and MAK-2 dynamic oscillations are restored in *Δham-11* germlings when communicating with wild-type germlings. The *ham-11* gene is restricted to the Sordariaceae, has no characterized orthologs, and encodes a plasma membrane protein with either one or two predicted N-terminal transmembrane domains and a long cytoplasmic C-terminal tail (Fischer et al., 2019). HAM-11 is phosphorylated in a MAK-2-dependent manner (Jonkers et al., 2014). Recently, a genetic interaction between *ham-11* and *doc-1/doc-2* was identified that regulates reinforcement of MAK-2 signaling during chemotropic interactions (Fischer et al., 2019).

The *Δham-12* mutant has a reduced frequency of germling communication and fusion compared to wild-type cells, and *Δham-12* germlings communicate with wild-type germlings at an equivalently reduced frequency (Leeder et al., 2013). The *ham-12* gene is conserved among the filamentous Ascomycetes (Pezizomycotina), and is predicted to encode a protein of unknown function with one transmembrane domain.

The *Δham-13* mutant has reduced frequency of germling communication and fusion as compared to wild-type cells. The HAM-13 protein was discovered in an Affinity Purification Mass Spectrometry (AP-MS) experiment to identify proteins that physically interact with MAK-2 (Dettmann et al., 2014). The HAM-13 protein likely interacts indirectly with MAK-2 via other members of the MAK-2 pathway complex. HAM-13 is also required for full phosphorylation of MAK-2.

The HAM-14 protein was identified as a phosphorylation target of MAK-2 and it is necessary for germling fusion, but not hyphal fusion (Jonkers et al., 2014, 2016). The HAM-14 protein physically interacts with MAK-2 and MEK-2 via co-immunoprecipitation, but was not observed to co-localize with fluorescently tagged MAK-2 (Jonkers et al., 2016). These observations led to the hypothesis that HAM-14 may facilitate the formation of MAK-2-pathway complexes, but HAM-14 is not involved in the oscillatory recruitment of MAK-2-pathway complexes during chemotropic interactions (Jonkers et al., 2016).

### Proteins That Physically Interact With the MAK-2 Complex

The YPK-1, CKA, CKB-1, and CDC-25 proteins were all identified as having a weak physical interaction with the MAK-2 complex via AP-MS, and these proteins were also shown to be required for full phosphorylation of MAK-2 (Dettmann et al., 2014). The *Δypk-1, Δcka, Δckb-1*, and *Δcdc-25* mutants all show a reduced frequency of communication and reduced levels of phosphorylated MAK-2 as compared to wild-type cells (Dettmann et al., 2014). *ypk1* encodes a serine/threonine protein kinase in *S. cerevisiae* that phosphorylates regulators of sphingolipid-biosynthesis, which is an essential structural component of cellular membranes (Roelants et al., 2011). The *cka* and *ckb-1* genes encode the two subunits of the casein-kinase-2 (CK2) heterodimer; CK2 is conserved across eukaryotes and mediates a broad diversity of biological processes. In *N. crassa*, CK2 is known to be an important component of the circadian clock, specifically in response to temperature (He et al., 2006; Mehra et al., 2009). The *cdc-25* gene encodes a conserved guanine nucleotide exchange factor. In addition to CDC-25 affecting the phosphorylation of MAK-2, CDC-25 is phosphorylated in a MAK-2-dependent manner (Jonkers et al., 2014). In *S. cerevisiae*, Cdc25p is required for progression through the G1 phase of the mitotic cell cycle and indirectly regulates adenylate cyclase via Ras1p and Ras2p (Broek et al., 1987).

### Transcriptional Targets of ADV-1

The genes *ncu04645*, *ncu04487*, *ncu05836*, and *ncu05916* were identified as targets of a communication-activated transcription factor protein, ADV-1 (Fischer et al., 2018). The NCU04645 protein is orthologous to IDC4 in *P. anserina* (Gautier et al., 2018) and has a putative C-terminal AIM24-like domain, which is involved in coordinating the MICOS protein complex on the mitochondrial membrane. However, neither NCU04645 nor IDC4 contain a predicted mitochondrial targeting signal sequence. Consistent with this prediction is the observation that IDC4-mCherry localizes to the cytosol (Gautier et al., 2018). The *N. crassa*ΔNCU04645 mutant is completely defective at germling communication, while the *Δidc4* mutant in *P. anserina* is defective at fruiting body development and suppresses the crippled growth phenotype (Gautier et al., 2018). The ΔNCU04487, ΔNCU05836, and ΔNCU05916 mutants all showed a significantly reduced frequency of germling communication as compared to wild-type (Fischer et al., 2018). NCU04487 encodes a protein with a predicted C-terminal transmembrane domain, NCU05836 is orthologous to the putative ER mannosidase Mnl2p in *S. cerevisiae* (Nakatsukasa et al., 2001), and NCU05916 is a putative alpha-1,3-mannosyltransferase with homology to the virulence protein CMT1 in *Cryptococcus neoformans* (Sommer et al., 2003).

### *pkr-*1

The *pkr-1* gene was identified in a screen for mutants defective at germling communication and female sexual development (Fu et al., 2011). The *pkr-1* gene is conserved among fungi and encodes a putative ER membrane protein. In *S. cerevisiae*, Pkr1p is an important assembly factor involved in the formation of the V-ATPase in the membrane of the ER (Davis-Kaplan et al., 2006).

### *idc-*3

The *idc-3* gene was identified in a screen for *P. anserina* mutants that suppressed a crippled growth phenotype (Lalucque et al., 2016). In addition to crippled growth suppression, the *P. anserina Δidc-3* mutant is defective at cell fusion and female sexual development. The *idc-3* homologous gene from the plant endophyte *E. festucae* was sufficient to complement the phenotype of the *P. anserina Δidc-3* mutant (Lalucque et al., 2016), indicating that *idc-3* function is likely to be broadly conserved among the filamentous Ascomycete species. The IDC-3 protein also has two conserved cysteine residues that may participate in NOX-related ROS signaling (Lalucque et al., 2016).

### *lao-*1

The *lao-1* gene encodes a L-ascorbate-oxidase-like laccase, and the *Δlao-1* mutant was shown to be completely defective at germling fusion (Lichius, 2010). The LAO-1 protein has multiple predicted copper-oxidase domains and there are no biochemically characterized orthologs of *lao-1*.

### *arg-15, spr-7*, and *nik-*2

The *arg-15*, *spr-7*, and *nik-2* genes were identified in a genome wide association screen for genes associated with communication frequency across a genetic population of *N. crassa* wild isolate strains (Palma-Guerrero et al., 2013). The *Δarg-15* mutant has a reduced frequency of germling communication compared to wild-type, while the *Δspr-7* and *Δnik-2* mutants have an increased frequency of germling communication compared to wild-type (Palma-Guerrero et al., 2013). The *arg-15* gene encodes a putative acetylornithine-glutamate transacetylase that is orthologous to Dug2p in *S. cerevisiae*, which is involved in degrading glutathione and other peptides containing a gamma-glu-X (Kaur et al., 2012). Glutathione is an antioxidant that reacts with ROS for detoxification in cells experiencing oxidative stress (Pócsi et al., 2004). The *spr-7* gene encodes a putative secreted subtilisin-like serine protease, with no clear orthologs that have been characterized. Given the hyper-fusion phenotype of the *Δspr-7* mutant it is tempting to speculate that the SPR-7 may negatively regulate an extracellular signaling peptide. However, subtilisin-like serine proteases encompass a broad diversity of enzymes (Siezen and Leunissen, 2008), and it is unclear what type of subtilisin-like protease is SPR-7. The *nik-2* gene encodes a histidine kinase with no clear orthologs that have been characterized. However, in *S. cerevisiae* and other fungi, histidine kinases function upstream of the osmotic stress MAPK pathway (Zhao et al., 2007).

## Conclusion and Summary

**FIGURE 3:**
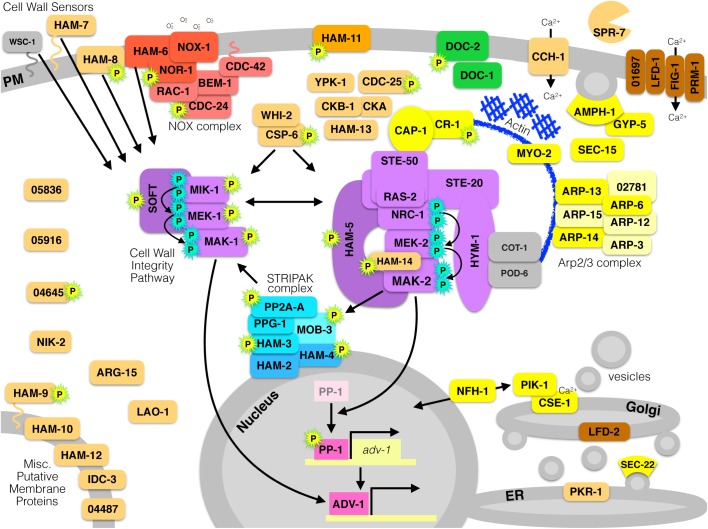
All currently known proteins required for germling communication and fusion in *N. crassa.* Proteins are color-coded by function. **Red** proteins are components of the NOX complex that produces superoxide by reducing NADPH and oxidizing molecular oxygen. **Purple** proteins make up two different MAPK pathways; the Cell Wall Integrity/MAK-1 pathway, or the MAK-2 signal response pathway. **Yellow** proteins are involved in actin dynamics, vesicle trafficking, endocytosis/exocytosis, or secretion. **Green** proteins are involved in long-distance non-self recognition. **Blue** proteins compose the STRIPAK complex. **Pink** proteins are transcription factors. **Brown** proteins are involved in membrane fusion. **Orange** proteins are uncharacterized or have an unknown function. **Gray** proteins are associated and relevant, but are dispensable for communication or fusion. Proteins with a **cyan-P** are phosphorylated as part of a MAPK cascade, and proteins with a **yellow-P** with a green outline are phosphorylated in a MAK-2-dependent manner.

The mechanisms that mediate communication and fusion in *N. crassa* are complex and integrate components of several pathways, which are depicted in Figure 3. Several factors influence crosstalk between the MAK-2 pathway, the MAK-1 pathway, and the STRIPAK complex. The broadly conserved regulatory proteins RAC-1, BEM-1, CDC-24, and CDC-42 have been directly implicated as part of the NOX complex in the filamentous Ascomycetes, but it is likely that these proteins also regulate chemotropic growth by influencing actin dynamics and the MAK-2 pathway. Several genes represent various aspects of the canonical ER-Golgi secretion pathway, for which actin dynamics are critically important. Calcium plays an important role in mediating both communication and cell fusion. Lastly, there are several genes that remain uncharacterized, except for their necessity during cell communication in *N. crassa.* Future work will elucidate the functions of these uncharacterized genes and further illuminate the complex signaling network that mediates somatic cell-to-cell communication in filamentous fungi.

## Author Contributions

MF and NLG wrote and edited the review. MF produced the figures.

## Conflict of Interest Statement

The authors declare that the research was conducted in the absence of any commercial or financial relationships that could be construed as a potential conflict of interest. The reviewer AL declared a past co-authorship with one of the authors NLG to the handling Editor.
